# Response—Corruption, Trust, and Professional Regulation

**DOI:** 10.1007/s11673-021-10149-5

**Published:** 2021-12-02

**Authors:** Kathleen Montgomery

**Affiliations:** 1 Professor Emerita of Organizations and Management, School of Business, University of California, Riverside, Riverside, CA 92521 USA; 2 Sydney Health Ethics, University of Sydney, Sydney, Australia

**Keywords:** Trust, Scientific misconduct, Research integrity, Stakeholders, Professional regulation

## Abstract

In their 2018 article in the *Cambridge Quarterly of Healthcare Ethics*, Little, Lipworth, and Kerridge unpack the concept of corruption and clarify the mechanisms that foster corruption and allow it to persist, noting that organizations are “corruptogenic.” To address the “so-what” question, I draw on research about trust and trustworthiness, emphasizing that a person’s well-being and sense of security require trust to be present at both the individual and organizational levels—which is not possible in an environment where corruption and misconduct prevail. I highlight similarities in Little et al.’s framing of corruption to the persistent problem of scientific misconduct in research and publishing. I acknowledge the challenges in stemming corruption in science and medicine and conclude with a discussion about the need to reinvigorate a web of stakeholders to actively engage in professional regulation.

## Defining Corruption

In their article, “An archeology of corruption in medicine,” Little et al. (2018) do a superb job unpacking the concept of corruption and clarifying the individual and institutional mechanisms that foster corruption and allow it to persist. This is no small feat, as “corruption” is one of those ubiquitous words, like “trust” that is tossed around with the assumption that readers are all on the same page about what the concept means. Little et al. show us the need for precision in defining the concept, in order to explore ways to deal with its potential presence and impact. All too often, this fundamental step in defining a complex concept is skipped, resulting in policy interventions that lead only to frustration.

The authors begin by highlighting the “what” of corruption—namely, the essential ingredients of corruption in a contemporary sense. They explain that corruption is (a) an action with a set of agents in an exchange that disadvantages other agents, (b) behaviour that is outside regulatory standards and social norms, and (c) behaviour that depends on institutions and organizations for credibility and authority.

Next, they unpack the “how” of corruption, by noting that institutions are “corruptogenic” in that they foster latent opportunities for someone to manipulate relationships to one’s advantage, especially for those individuals whose habitus, or personality traits, make them amenable to corrupt behaviour. The authors explain that, while corruption can occur insidiously within a single institution, perhaps its more profound impact is when corruption crosses institutional or organizational boundaries, especially when the corrupting agents are powerful.

Little et al. apply insights from this framework to examine degrees of corruption in relationships between medicine and the pharmaceutical industry. With reference to well publicized cases of corruption, the authors describe a continuum of corruption—from the most egregious, intentional acts of personal aggrandizement at one extreme, to the middle range of laissez-faire attitudes or willful ignorance about coworkers’ corruption, to whistle-blowing as active resistance to corruption at the other extreme. They provide a thoughtful discussion of the nuances of “beholden-bias” that can trigger behavior in the center range of the corruption continuum. This gift-exchange phenomenon can arise through pharma’s influence on medical decision-making that begins with small gifts of pens and notepads and escalates into sponsorship of clinical trials and educational programmes.

## The So-What Question: Damage to Trust Relationships

At the heart of Little et al.’s article is the “so-what” question. Little and colleagues explain how corruption can cause psychological harm, generating anger in stakeholders who have operated on assumptions of beneficence, and damaging stakeholders’ trust in individuals and institutions. This is borne out in a recent Pew Research Center survey investigating elements of declining trust in the medical profession, which reported that 50 per cent of respondents believe professional misconduct is a “very big” or “moderately big” problem (Pew Research Center 2019). Indeed, declining trust in medical professionals, in general, has been widely reported in recent decades, and the downward trend in trust level continues (Balaban 2020).

When trust is damaged, cooperation suffers. One way to examine the central importance of trust in human service professionals is to consider the situation of individuals facing extreme events (such as a natural disaster, an industrial accident, or a life-threatening illness). Montgomery et al. (2008) explain that “imposed vulnerability” caused by the extreme event itself is exacerbated by having to rely on and cooperate with professional surrogates—e.g., rescuers and medical experts—to help restore a sense of security. The authors point out that, when needy individuals decide to place their well-being in the hands of surrogates, it generates “elective vulnerability” that could further imperil the individuals if the surrogates turn out to be unreliable.

The authors highlight the centrality of trust to explain why such individuals would make this risky choice anyway. They begin with a generic definition of trust:

The willingness of an individual (The Truster) to be vulnerable to the actions of another party (The Trusted) on a matter of importance to the Truster, based on the expectation that the Trusted party will behave in a way that doesn’t take advantage of the Truster, even when the Trusted’s behavior can’t be monitored or controlled. (625)

Importantly, this definition of trust stands in striking contrast to a main feature of Little et al.’s definition of corruption as an action that *disadvantages* other agents.

Montgomery and colleagues clarify that trust is only possible when professional surrogates act in a trustworthy way (i.e., with competence, benevolence, and integrity that includes honesty, fairness, and transparency), as well as when they represent organizations and agencies that also are deemed to be trustworthy. This illustrates the essential link between professionals and organizations, another key element in Little et al.’s concept of corruption that emphasizes the role of institutional credibility.

Thus, trust cannot exist in an atmosphere of corruption, as defined by Little and colleagues. And, without trust, there will be no willingness to cooperate with another. In the example of extreme events, a lack of trust in surrogates would lead to a resistance to cooperate with rescue efforts, resulting in a prolonged state of insecurity. In a more everyday example, a lack of trust in medical professionals can lead to failure of patients to follow recommended treatment protocols, thereby risking their health. And today, persistent mistrust of the medical profession and science in general, and the use of vaccines in particular, is thought to be a strong factor in public resistance to the COVID-19 vaccine, likely prolonging the pandemic (see the recent study reported by Johns Hopkins University 2021).

## Corruption in Academic Research and Publishing

In this section, I trace the striking parallels between corruption in academic research—often referred to as “scientific misconduct”—and Little et al.’s treatment of corruption in biomedicine and increasingly entangled relationships with pharma. Parallels include the frustrating challenges both to defining the concept and to quelling its persistence.

Montgomery and Oliver (2008) trace the shifts over time in how scientific misconduct has been defined and policed. They observe that prior to the mid-1970s, scientific conduct was assumed to abide by the Mertonian norms of universalism, communalism, disinterestedness, and organized scepticism (Merton 1973). The primary governance model was professional self-regulation, and oversight remained relatively passive. The inadequacy of this passive model became apparent in the face of widely publicized reports of fraudulent research funded by the U.S. government (Broad 1981; Culliton 1983).

Taking a more active approach to the problem, the U.S. Public Health Service issued a definition of “scientific misconduct” as.

Plagiarism, fabrication, or falsification of data, with the deliberate intent to deceive or mislead. It does not include honest error or honest differences in interpretation or judgments of data. (U.S. Public Health Service 1986)

This definition aligns closely with the definition of corruption proposed by Little and colleagues; namely, scientific misconduct is behaviour that disadvantages other agents (e.g., other scientists whose research may be plagiarized or unwitting users of falsified data); that reflects a violation of relevant social norms and regulatory standards (e.g., Mertonian norms of science and standards of intellectual honesty); and that relies on institutions and organizations for credibility (e.g., universities and research centres where the research is conducted and official venues where the research is presented and published).

Beyond questionable data practices listed in the above definition, the concept of scientific misconduct was augmented to include abuse in human subjects research (such as in the Tuskegee syphilis studies and the New York cancer experiments on indigent elderly [Faden and Beauchamp 1986]). In what is known as the “informed consent movement,” the National Commission for the Protection of Human Subjects (1979) issued guidelines that included failure to follow human subjects protections, including informed consent, as a serious form of scientific misconduct.

Responsibility for rooting out the corruption of scientific misconduct shifted from professional self-regulation to a governance model of administrative oversight, whereby federal granting agencies threatened to withhold funding from universities and scientists who were found to engage in questionable research practices and/or who did not comply with proper human subjects protections. This somewhat punitive approach was short-lived. Many scientists and their universities found such administrative oversight to be onerous and in violation of professional expectations for self-regulation. Moreover, reports of scientific misconduct continued to appear in the media, suggesting that this approach was not accomplishing its goal of stopping corruption in the research process.

By the end of the twentieth century, a more positive approach was being inaugurated across universities and federal agencies, with a shift in terminology from “scientific misconduct” to “responsible conduct of research” (RCR) or “research integrity,” defined as.

Intellectual honesty in proposing, performing, and reporting research; accuracy in representing contributions to research proposals and reports; fairness in peer review; collegiality in scientific interactions, including communications and sharing of resources; transparency in conflicts of interest or potential conflicts of interest; humane care of animals in the conduct of research; adherence to the mutual responsibilities between investigators and their research teams. (Institute of Medicine/National Research Council 2002)

Not surprisingly, this definition was seen primarily as an elaboration on Merton’s norms of universalism, communalism, disinterestedness, and organized scepticism—representing a return to the ideals of professional self-regulation. To advance this positive approach, universities incorporated ethics training into their educational programmes, designed to inculcate research integrity practices in science trainees. Professional associations beefed up their codes of ethics. These joint efforts were built on the belief that *explicit* guidelines about responsible conduct of research would go a long way to reduce scientific misconduct, recognizing that Merton’s *implicit* norms of scientific conduct had obviously been insufficient.

Determining the effectiveness of these efforts is not straight forward and data are hard to interpret. For example, the U.S. Office of Research Integrity (ORI) provides case summaries of investigations undertaken by that department in response to claims of scientific misconduct. Trend data in the last ten years suggest that the number of investigations has increased (U.S. Office of Research Integrity 2021). This figure could reflect heightened awareness and subsequent reporting activity, rather than an actual increase in misconduct behaviour. But it also may not fully reflect the extent of scientific misconduct because most cases of scientific misconduct never reach the level of ORI surveillance, as they often remain unrecognized and/or unreported. These instances likely fall in the middle range of the corruption continuum proposed by Little and colleagues that includes laissez-faire attitudes or wilful ignorance.

Another view into the persistence of corruption in science is offered by studies of retractions of published scientific research. Again, data are hard to interpret. Montgomery and Oliver (2017) examine the retraction process for papers that have been found to contain “false science” or “bad data” (papers with data that have been falsified or fabricated or which contain error). These authors cite reports that the rate of retractions spiked in recent decades (van Noorden 2011; Furman et al. 2012) and that over 60 per cent of retracted articles on drug studies were pulled because of bad data, either falsification or error, rather than plagiarism (Samp et al. 2012). In a recent examination of a large data set of retractions, Brainard and You (2018) acknowledge that it is not clear whether suspect papers are becoming more common or whether journals are just getting better at recognizing false science and taking action.

Another parallel to the framing by Little et al. appears in suggestions about the motivation for corruption. Little et al. explore the potential that a “beholden-bias” can infect professional decisions, as a result of implicit pressures from the gift-exchange phenomenon between pharma and biomedicine. Similarly, the reward systems in scientific research and publishing generate implicit pressures, fuelling an atmosphere of tolerance for potentially corrupt behaviour as a means to academic achievement.

## Stemming Corruption in Science and Medicine: It Takes a Village

Little and colleagues conclude by discussing a variety of efforts that have been undertaken to quell corruption, although they lament the difficulty in stopping a determined person from using the good name of an organization for personal gain.

As previewed here, the arsenal of approaches to stemming corruption can range from explicit articulation of appropriate behavioural norms and professional codes of ethics, to training and mentoring programmes, to requirements for transparency and accountability, to investigations of wrongdoing, to imposition of sanctions. This panoply of approaches requires cooperation—and, yes, trustworthiness—among key stakeholders in biomedicine and science, who ideally have a shared interest in ensuring that the biomedical research on which they rely in accurate and unbiased.

All too often, this goal is not achieved, however. In part, this is because there is an incomplete recognition of the varied stakeholders who are in a position to facilitate oversight, as responsible members of the scientific community. Leahey and Montgomery (2011) highlight the importance of attending to the range of stakeholders and their relationships with one another in regulating professional conduct. They argue that a thorough recognition of the web of stakeholders involved in professional regulation (who therefore carry the promise of reduction in corruption in biomedicine and medical research) enables joint efforts across levels of regulation and professional groups and bodies.

To illustrate using the model of a scientist engaged in biomedical research, at the heart of the web is the scientist, who is personally responsible for the conduct of research through self-regulation of his or her own behaviour, developed through norms of scientific integrity inculcated during training.

Should this personal self-regulation be ineffective (perhaps because of an individual’s habitus that tends toward corrupt behaviour and/or other irresistible pressures toward misconduct), the next level of stakeholders needs to step in. These stakeholders have several opportunities to detect misconduct and quell corruption because of their *direct* relationship with the research process—as collaborators and trainees working together on the project; as evaluators of a proposed research project (i.e., funding bodies and IRB committees); or as reviewers of a completed research project (i.e., journal editors and peer referees). It is at this level (a) that expectations for data accuracy can be instilled in the lab; (b) that requirements for responsible treatment of human subjects can be imposed; and (c) that full disclosure of potential funding conflicts of interest can be ensured. Their privileged position vis-à-vis the research itself places a substantial moral imperative on stakeholders at this level to act in concert in order to thwart misconduct and corruption before it spreads. This can be thought of as the primary line of professional regulatory defence against corruption, with the goal of making the profession and their organizations and institutions less corruptogenic.

The next level of stakeholders is composed of those with an *indirect* relationship to the scientist—universities and research centres, professional societies, accrediting bodies, and journal policy committees. Stakeholders at this level typically are not connected to a particular research project, yet they are in a strong position to foster appropriate research conduct throughout the profession using a variety of mechanisms. These mechanisms include providing training and continuing education programmes, establishing formal codes of ethics, overseeing implementation of institutional policies and practices, and formulating publishing guidelines. Thus, stakeholders at this level have a strong obligation at the organizational and institutional level to investigate claims of misconduct and corruption, and to impose sanctions when justified. This again demonstrates the critical importance that the professional community work together to form a higher level of defence against corruption and to mitigate corruptogenic opportunities.

The most removed level of stakeholders includes those with only a *distant* relationship to the scientist and a particular research project—government leaders and policymakers, the general public and citizen advocacy groups, and the media. We have seen that, when the more direct lines of defence are not sufficient to stem misconduct and corruption, stakeholders at this level may weigh in. While responses from stakeholders at this level may be heavy handed and punitive and may overstep professional expectations of autonomy, history has shown that such intervention may be necessary. A classic example is the 1972 Associated Press exposé of the Tuskegee syphilis research of Black men in Alabama (Heller 1972), ending a decades-long abuse of unwitting patients in a study conducted by the U.S. Public Health Service.

Note that pharma and other commercial industries are not included in the set of stakeholders in the regulatory process. This is because such industries, while part of the broad organizational field of biomedicine, do not function in the role of regulators. Instead, they present challenges to the web of stakeholders committed to appropriate professional behaviour by generating external pressures that may conflict with professional norms and scientific goals. Moreover, relationships with pharma and other commercial industries are but one source of pressures that can lead scientists down the path of misconduct.

Indeed, dealing effectively with conflicting external pressures is a herculean task, and there is no magic bullet. At best, we can follow Little and colleagues’ lead by, first, articulating what is meant by corruption and how it may infest corruptogenic institutions and organizations. With this knowledge, we can then examine similar situations where professional corruption and misconduct have been reported, along with interventions that have been undertaken to deal with the problem, albeit with only moderate success. More work is needed, and it requires an ongoing commitment to preserving the integrity of the scientific endeavour. To that end, in this essay I have emphasized the centrality of trust and cooperation among a web of motivated professional stakeholders, with the promise of developing a comprehensive, joint strategy to minimize corruption itself and cleanse their institutions of corruptogenic opportunities. This strategy ideally would include training in the obligations and responsibilities of all stakeholders in the professional regulatory web, not just for those actually engaged in the research.
